# An Air-well sparging minifermenter system for high-throughput protein production

**DOI:** 10.1186/s12934-014-0132-1

**Published:** 2014-09-14

**Authors:** Cecilia Deantonio, Valentina Sedini, Patrizia Cesaro, Fabio Quasso, Diego Cotella, Francesca Persichetti, Claudio Santoro, Daniele Sblattero

**Affiliations:** Department of Health Sciences and Interdisciplinary Research Center on Autoimmune Diseases (IRCAD), University of Eastern Piedmont “Amedeo Avogadro”, Via Solaroli 17, 28100 Novara, Italy; Department of Sciences and Innovation, University of Eastern Piedmont “Amedeo Avogadro”, Alessandria, Italy

**Keywords:** High-Throughput Protein Production, Shake-flask, *E.coli*, Air-sparging, Minifermenter

## Abstract

**Background:**

Over the last few years High-Throughput Protein Production (HTPP) has played a crucial role for functional proteomics. High-quality, high yield and fast recombinant protein production are critical for new HTPP technologies. *Escherichia coli* is usually the expression system of choice in protein production thanks to its fast growth, ease of handling and high yields of protein produced. Even though shake-flask cultures are widely used, there is an increasing need for easy to handle, lab scale, high throughput systems.

**Results:**

In this article we described a novel minifermenter system suitable for HTPP. The Air-Well minifermenter system is made by a homogeneous air sparging device that includes an air diffusion system, and a stainless steel 96 needle plate integrated with a 96 deep well plate where cultures take place. This system provides aeration to achieve higher optical density growth compared to classical shaking growth without the decrease in pH value and bacterial viability. Moreover the yield of recombinant protein is up to 3-fold higher with a considerable improvement in the amount of full length proteins.

**Conclusions:**

High throughput production of hundreds of proteins in parallel can be obtained sparging air in a continuous and controlled manner. The system used is modular and can be easily modified and scaled up to meet the demands for HTPP.

## Background

The increasing amount of data derived from genome scale studies has forced the implementation of protein expression pipelines that support high throughput (HT) screening and testing of cloned gene products [1,2]. Typically, these pipelines combine batteries of cultivation devices eventually integrated with fluidic-handling and/or robotic apparatus for automated processes. Tubes or shake flasks have been the standard lab-scale cultivation devices for decades and have been used in industry and academia to grow a wide range of microorganisms as well as mammalian cells. They are easy to operate and can be designed for automated monitoring of cultivation parameters such as pH and dissolved oxygen. However, they are unfit for HT operations as their scale-up possibilities are limited to a few dozen parallelized cultures. Thus, there has been an intense effort to design cultivation apparatus that allows growth of hundreds to thousands of cultures in parallel. This goal is typically achieved by two means: reducing the working volume of cultures to milliliter-scale or below, and using bacteria as expression hosts [3].

Deep-well microtiter plates are regularly employed as easy-to-use miniaturized minifermenters. They allow working with multiples of 96 cultures simultaneously. The biomass and the amount of recombinant protein obtained by milliliter-scale cultures are satisfactory for most research purposes and allow selection of protein candidates for scale-up production and expression. Fast cultivation, easy handling and well-characterized genetics make *Escherichia coli* one of the most widely used hosts for the production of heterologous proteins. However, even in such a microorganism, the production of recombinant proteins at high levels and biologically active form is not guaranteed, being influenced by the physico-chemical properties of each protein and by cultivation conditions. In particular, when operating with microtiter-based systems, nutrient consumption and aeration play a major role [4]. Typically bacterial cultures are carried out in batch mode by shaking or stirring a fixed volume of rich medium [5]. Unfortunately, at high cell densities, nutrients can become limiting and lead to carbon catabolite repression, consequent growth impairment and reduced production of the desired protein. In addition, the small specific mass transfer area of multiwell plates limits oxygen diffusion, leading to stress responses and medium acidification [6].

To circumvent these problems, several strategies have been proposed regarding medium composition, on-line feeding and aeration [7]. For instance, it has been shown that substituting glycerol for glucose in bacterial broth overcomes overflow metabolism and the accumulation of fermentation metabolites [8]. Furthermore, new media formulations have been proposed that allow tight regulation of the expression of recombinant protein by auto-induction, thus minimizing off-line interventions during culture growth [9]. Several small-scale systems have been designed that are suitable for parallelized cultivations in fed-batch mode [10,11]. In fed-batch cultures one substrate solution can be continuously maintained at limiting concentrations in order to control growth and match the oxygen transfer rate [5]. Demanding constant control, fed-batch cultures are usually performed in bioreactors, where cultivation parameters and nutrient supply are highly automated [12]. One of the most effective technologies is the Robo-Lector platform [11], a microfermentation system in which bacterial growth and protein production are monitored using non-invasive on-line monitoring signals and can be coupled to downstream protein purification devices. However, despite medium and culture automation improvements, aeration remains the major cause of bacterial growth impairment and poor protein production [13]. Orbital shaking is the most used and cost-effective means to achieve aeration. The influence of shaking diameter, frequency and filling volume on gas–liquid mass transfer has been evaluated both theoretically and experimentally [14]. In practice, oxygen transfer capacity increases with increasing gas–liquid mass transfer area [6]. However, when operating with microtiter wells at standard shaking conditions (100–300 rev./min, shaking diameters 6–20 mm) the gas–liquid mass transfer area remains mainly constant. This is due to the effect of interfacial tension throughout the microtiter well [6]. Thus, the increase of oxygen transfer can be obtained only beyond a critical shaking frequency, when a critical centrifugal force overcomes the surface tension of the medium. On the other hand, shaking intensity cannot be increased beyond the point when liquid spillage occurs between adjacent wells.

In this paper we present a novel device that uses air sparging as an alternative to shaking for microbial cell cultivation in 96 deep-well plates. By comparative analyses we demonstrate that *E. coli* clones cultured in the novel apparatus grow faster and reach higher cell densities than by shake cultivation. Furthermore, the quantity and quality of the produced recombinant proteins are significantly improved.

## Results

### Overall features of the cultivation system

**Figure 1:**
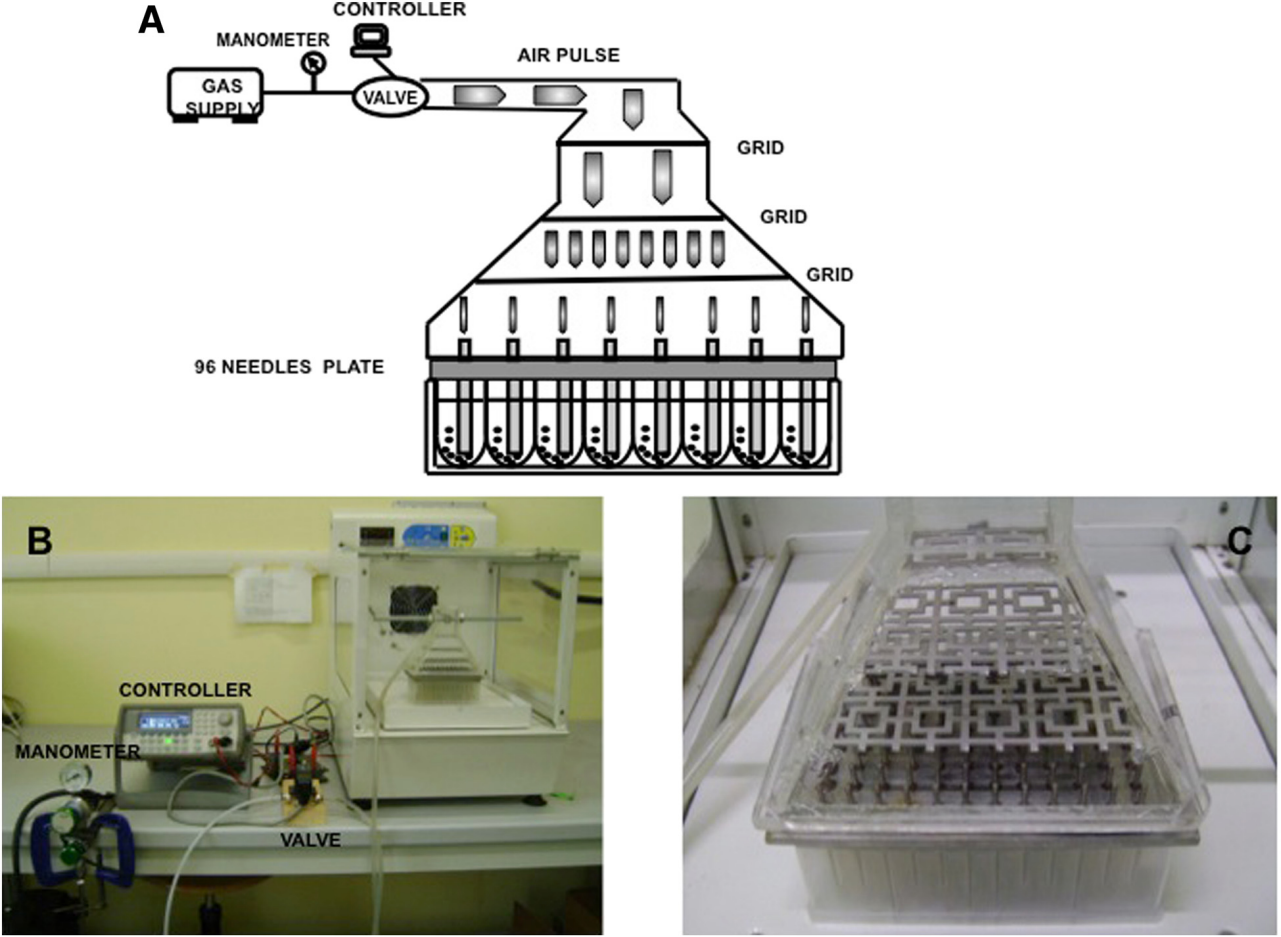
**Air-Well minifermenter scheme/layout. A)** schematic representation of the Air-Well minifermenter. **B)** the Air-Well minifermenter. **C)** Magnification of the Plexiglas bell with the grid used to equally split the air flux.

Forced by a need to express and screen several hundred recombinant proteins in *E. coli*, we designed a system to allow growth of multiples of 96 cultures, at milliliter-scale, in batch-mode with little instrumentation and lab-space requirements. The key feature of the system is that simultaneous agitation/aeration of cultures is provided through a simple air sparging device (Figure 1A,B; Air-Well minifermenter) powered by a low-pressure air supply. The core part of the device consists of two elements: a stainless steel plate holding 96 needles (96 needle plate) that is clamped to an air flow diffusing Plexiglas bell (see Materials and Methods for details). When assembled over a 2 ml deep-well microtiter plate (Figure 1B), a cultivation unit is obtained that is connected, singly or in multiple units, to an air flow source. After building a few prototypes of differing construction materials, needle width or shape, we finally opted for the following key characteristics: i) a thick stainless steel plate that could be repeatedly autoclaved, was not deformable and was sufficiently heavy to weigh on the multiwell plate; ii) 96 needles that fit inside each well to a depth of 1–1.5 mm from the bottom to prevent cell sedimentation and extend the time of gas–liquid mass exchange; iii) the presence of three grids inside the divergent bell to split air flux evenly to all 96 needles (Figure 1C). The Air-Well minifermenter is powered by pulsed low pressured air influx. The pulse period and pulse width are defined by a wave generator that controls a solenoid valve (for details see Material and Methods).

### Air-Well minifermenter design and parameters

**Figure 2:**
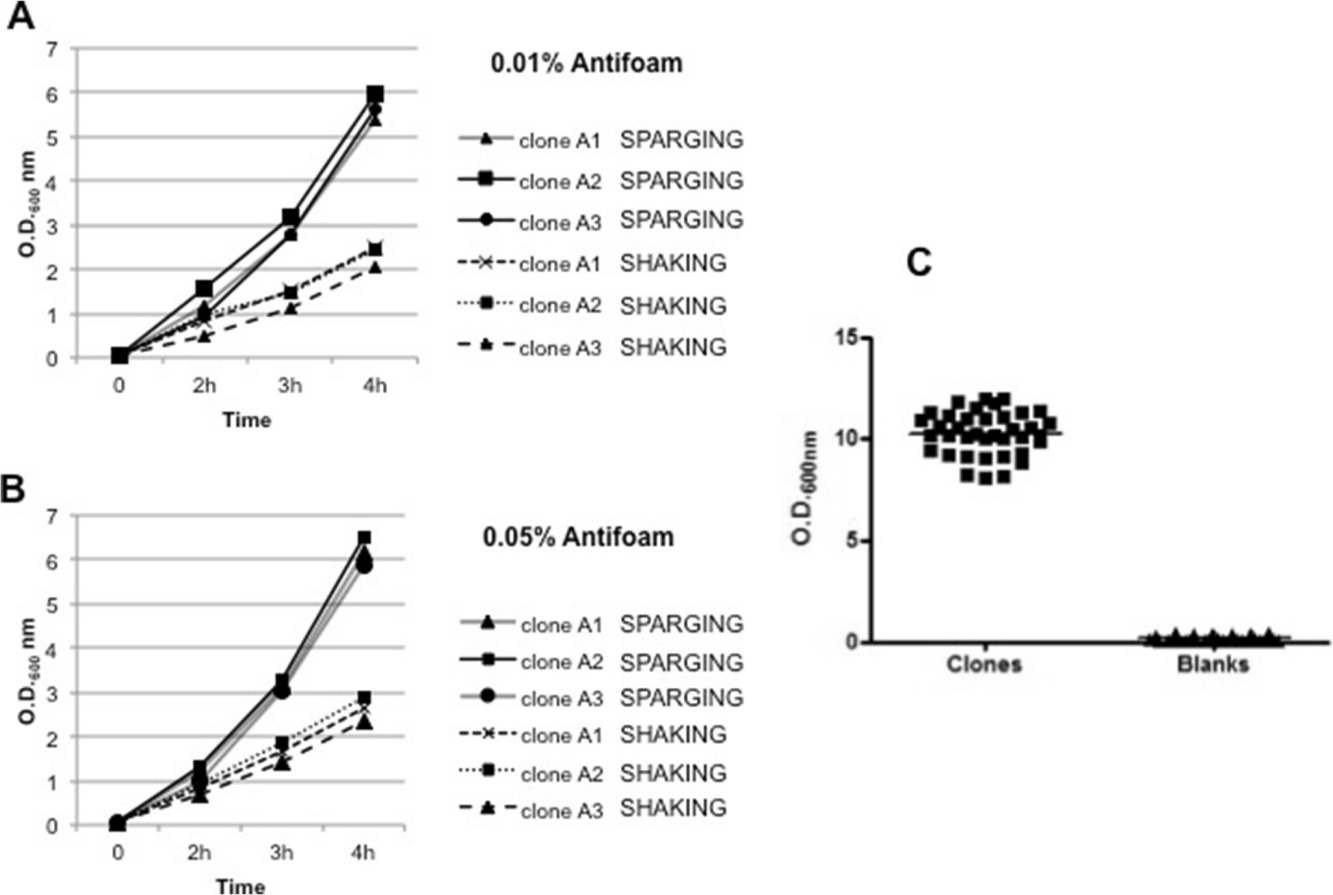
**Growth curves of clones with anti foam.** 3 different clones were grown in parallel in the presence of 0.01% **A)** or 0.05% **B)** antifoam in shaking or air sparging condition. **C)** Scatter plot of the O.D._600_ of 96 wells on a air sparging plate grown after an overnight in presence of 0.01% antifoam. Only 38 wells were inoculated with bacteria (clones) to check cross contamination in empty wells (blanks).

**Figure 3:**
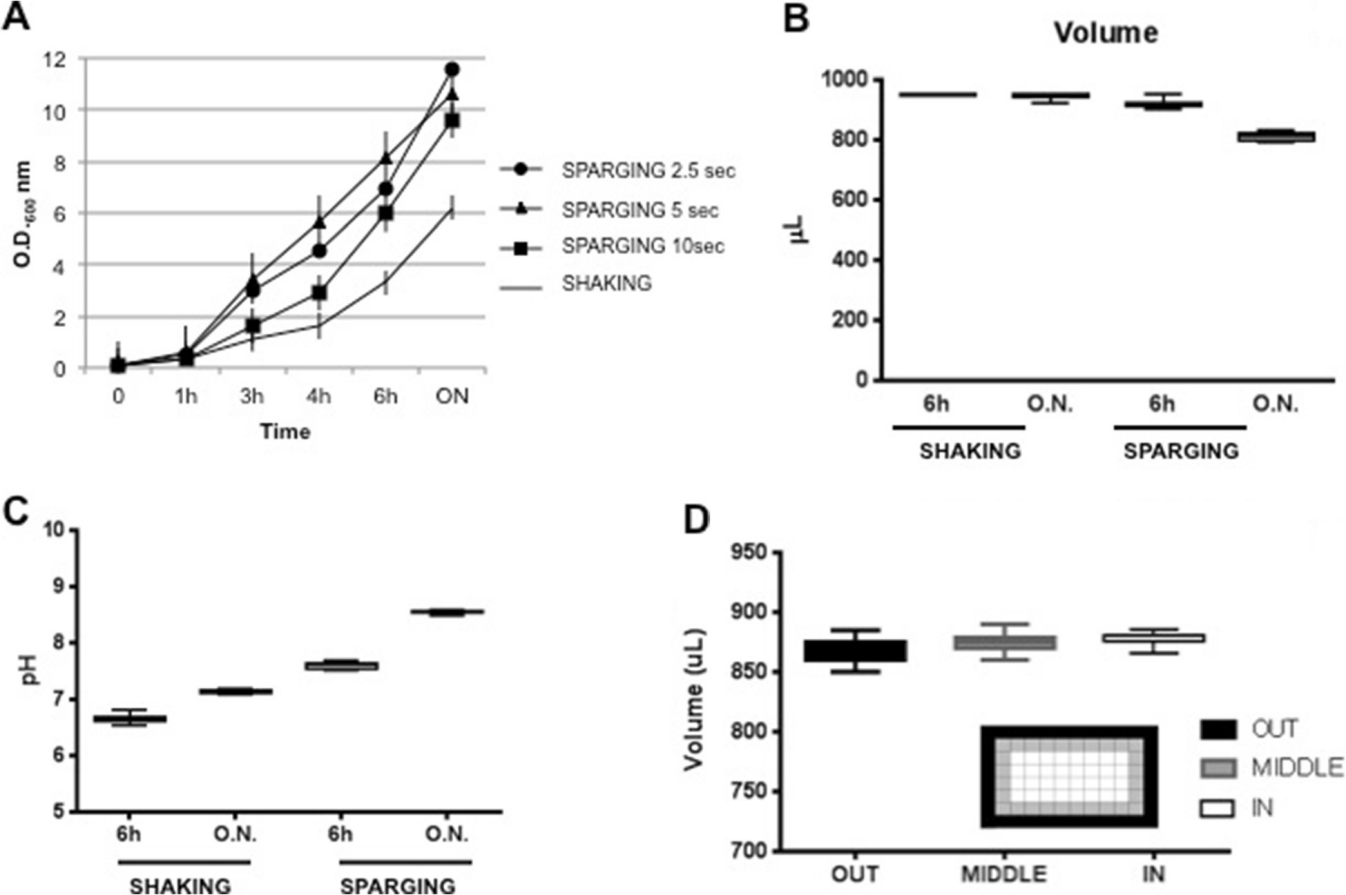
**Growth curves of clones with different air sparging periods. A)** Average growth curves of 24 clones grown by 3 different air sparging periods (SPARGING 2.5; 5; 10 sec) or by orbital shaking (SHAKING). O.D._600_ measured at 5 different times. **B)** Comparison of the remaining volumes of media after O.N. shaking or air sparging (period 5 sec) growth. **C)** pH of 24 wells evaluated after 6 h or O.N. in shaking and air sparging condition. **D)** Evaluation of evaporation differences between peripheral (OUT) central (MIDDLE) or internal (IN) wells within the plate (time 6 h; period 5 sec). Inlets represent plates and the wells analysed.

The major advantages of the Air-Well minifermenter are the following: the cultivation unit is stationary and up to 4 units (384 cultures) can be hosted in a small bench-top thermostatic cabinet (an example is shown in Figure 1); several Air-Well minifermenter units can be powered by a single air flow source, and considering the low pressure even a portable aquarium air pump might suffice; the air sparging into a deep-well plate increases oxygen transfer and avoids sedimentation of the bacteria cultures during the incubation; the use of autoinduction medium minimizes manual interventions during culture growth. However, when we first ran the system with continuous air influx, we observed a significant production of bubbles in all wells, a phenomenon that could favour cross-contamination and high evaporation of well content. We addressed this problem by two means. First, we decided to supply air influx as pulse waves based on the empirical observation that, though bubbles dissipated spontaneously, the process required 1–2 seconds to occur. This was accomplished by inserting a solenoid valve downstream of the air flow source and providing defined pulse parameters with a wave generator. Then we evaluated whether the inclusion of an antifoam reagent in the culture medium could accelerate the dissipation of bubbles without affecting bacterial growth. Three different bacterial clones were inoculated in autoinduction medium supplemented with 0.01% or 0.05% antifoam and grown in parallel, either by shaking (orbital diameter 2.5 cm and 200 rpm) or by pulsed air sparging (period 2.5 s, width 200 ms). OD_600_ values were measured at 2, 3 and 4 h after inoculation. The results demonstrated that the inclusion of antifoam in the medium did not affect bacterial growth at either concentration (Figure 2). We also verified that the presence of 0.01% antifoam in the medium was sufficient to avoid well to well cross contamination. We filled each well of a 96 deep-well plate with 1 ml of sterile antifoam containing medium and randomly inoculated 38 wells with different bacterial clones. After an overnight growth in air sparging mode (period 2.5 s, width 200 ms), the OD_600_ values of all 96 wells were measured. As shown in Figure 2C, none of the uninoculated wells were contaminated by the adjacent cultures. As it is likely that the frequency and width of air pulses influence bubble quantity and quality, as well as the growth of bacteria, we decided to optimize these parameters. We used four 96 deep-well plates, each one containing 24 wells filled with 1 ml of autoinduction medium (0.01% antifoam) and freshly inoculated with an identical clone. One plate was grown by orbital shaking at 200 rpm (orbital diameter 2.5 cm), the others by air sparging at fixed pulse width (200 ms) and three different pulse periods (10 s; 5 s; 2.5 s). Mean OD_600_ values were determined at 1, 3, 4 and 6 hours of growth or after overnight incubation (sample volume was adjusted to 1 mL before the measurements). As expected, air sparging significantly increases the growth rate of cells when compared to shaken cultures (Figure 3A). Although at first sight these results might indicate that the best condition to use is a pulse period of 2.5 s for an overnight culture, we also observed two effects that lowered the appeal of these conditions. The volume of overnight cultures grown with air sparging, at any pulse period, was significantly reduced (15-18%; Figure 3B). Moreover, the pH of these cultures rose above 8 (Figure 3C) indicating that microorganisms switched to peptide catabolism [12]. On the other hand, these results also showed that the biomass obtained after 6 hours of cultivation by air sparging, in particular at a pulse period of 5 s, was double that of shaken cultures, with acceptable variations in culture volume and pH (Figure 3B-C). To check if these variations are dependent on the position of the culture within the plate, we used a 96 deep-well plate containing all wells filled with 900 μl of autoinduction medium freshly inoculated with a single clone, grew it for 6 hours at 37°C (pulse period 5 sec) and then measured the culture volume of each well. The Plate was divided into three different zones (inlet of Figure 3D), peripheral, middle and central and as shown in Figure 3, we did not observe significant differences within these zone in terms of evaporation rate (Figure 3D). Based on these evidences, we decided to use a growth time of 6 hours and a pulse period of 5 sec to perform subsequent protein production experiments.

### Recombinant protein production

**Figure 4:**
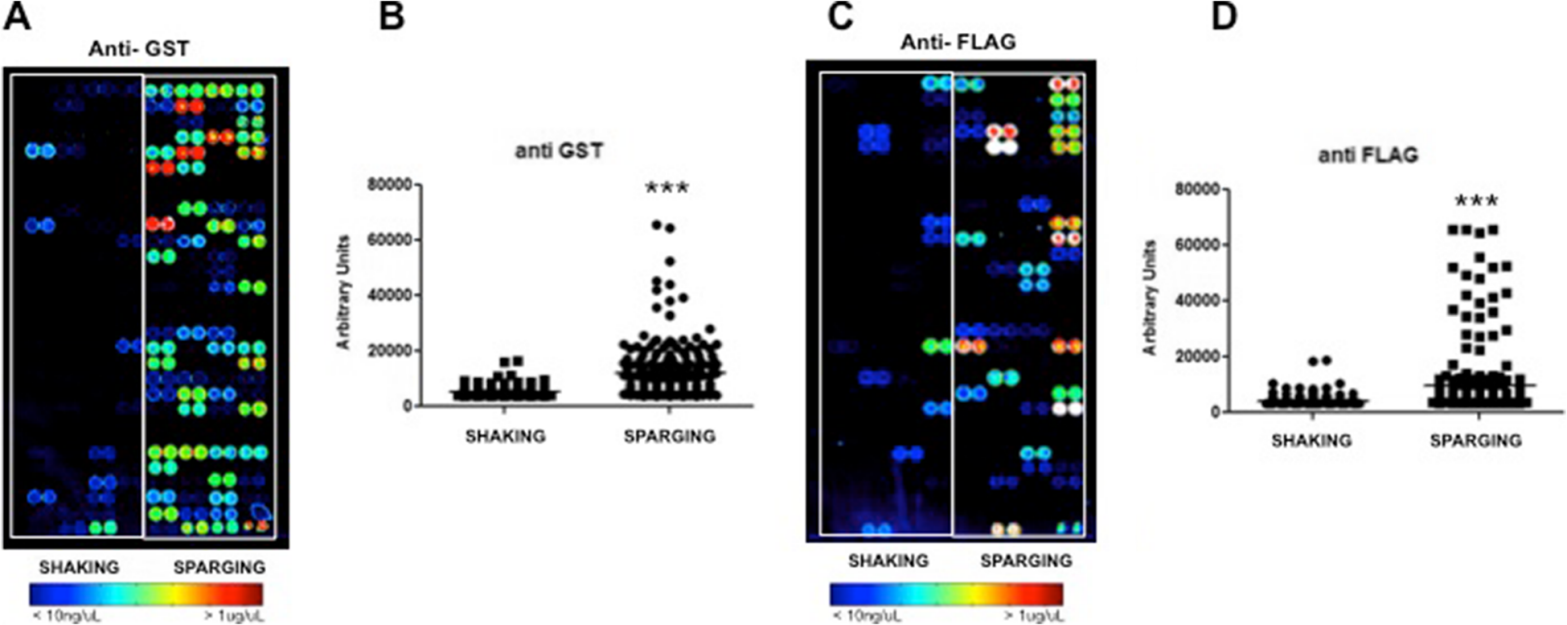
**Protein quality detection by microarray. A**-**C)** Images of proteins produced by orbital shaking (SHAKING) and air sparging (SPARGING) arrayed onto Whatman nitrocellulose FAST® slides and detected through anti-GST and anti-FLAG antibodies. **B**-**D)** Scatter plot of the fluorescent intensity quantification of the proteins revealed by anti-GST and anti-Flag antibodies (***P < 0.0001).

Encouraged by the above data, we wanted to verify whether the Air-Well minifermenter could offer any advantage in the production of recombinant proteins when compared to shaken microtiter plates. In particular, we wanted to carry out an experiment that reflected real conditions encountered in a screening process. To this end, we used random clones derived from a previous screen [15] and predicted, from DNA sequence data, to express different fusion proteins, all sharing GST and Flag-tag at their N- and C-termini, respectively. The presence of two different tags fused at both termini of a protein allows to detect it and to distinguish between any expressed product (detected by either antibodies) and full-length products (detected by both anti-GST and anti-Flag antibodies). Thus, we inoculated two deep-well plate replicates with 95 different clones in autoinduction medium. Parallel cultures were grown for 6 hours, one plate by air sparging (0.01% antifoam and pulse period 5 s) the other by orbital shaking (orbital diameter 2.5 cm and 200 rpm) [14]. The two plates were processed in parallel and the protein purification was performed as described in the Materials and Methods section [16]. The impact of the new cultivation system was evaluated by protein microarray analyses [15]. This method allows the analysis of hundreds of proteins simultaneously and is becoming a standard in HT screening processes. Furthermore, several array copies can be made with small amounts of protein and analysed by different means to get insights on protein status and properties [17]. After affinity purification 95 proteins produced from shaken or Air-Well minifermenter cultures were arrayed as duplets onto nitrocellulose slides and analysed with anti-GST antibody to evaluate the amount of recovered proteins. As shown in Figure 4, 66 out of 95 proteins (70%) from cultures grown by the Air-Well minifermenter were detected with the anti-GST antibody, while only 19 (20%) from shaken cultures (Figure 4A) could be detected.

This result indicates that the Air-Well minifermenter provides an overall significant advantage in the production of recombinant proteins (Figure 4B). To assess if the cultivation mode could influence the quality of expressed proteins as well, we analysed a protein array replica with an anti-Flag antibody in order to detect the full-length fusion proteins. As shown in Figure 4C, the majority of proteins (55%) obtained with the Air-Well minifermenter resulted full length. Thus we can conclude that in terms of amount and quality of the produced proteins, cultivation in the novel Air-Well minifermenter provides a significant advantage compared to the shaken mode (Figure 4B,D).

## Discussion

Microtiter plates are an established tool widely used in diagnostics because of the possibility of performing hundreds of reactions in parallel and at small scale [18]. For these reasons, microtiter plates have also been adopted by laboratories involved in high-throughput screening of recombinant expression clones. Several cultivation system based on the microtiter standard have been proposed, some featuring high level of automation and the possibility to monitor cultivation parameters [11]. In these systems, agitation and aeration of cultures are commonly achieved by orbital shaking of the plate. A limited oxygen transfer capacity is typical of any shaken device but in microtiter plates, in particular the 96 format or higher, there is an additional problem due to the interfacial tension that counteracts the centrifuge force provided by shaking [14]. Thus, microtiter plates have to be shaken at critical frequencies to guarantee oxygen transfer rates (OTRs) comparable to those obtained in shaken or stirred flasks [19] [6]. As a consequence, using standard lab-scale shakers, OTR cannot be improved by increasing shaking frequency without the risk of medium spillage and cross-contamination. The use of breathable membranes to seal wells is not a solution, since they significantly decrease oxygen transfer [20]. Because of these concerns, we designed a simple device, referred to as the Air-Well minifermenter, that could allow production of hundreds of recombinant proteins at a scale sufficient for screening needs. The device is based on the 96 deep-well microtiter standard, and its key feature is the use of air sparging as a mean to agitate/aerate bacterial cultures. Gas sparging has been used for decades in industrial reactors as an efficient method to increase gas–liquid mass transfer surface area, but its use in cell cultivation is limited to few examples of miniaturized cultivation devices. Doig et al. have reported a microtiter-based bubble reactor in which air is pumped through a porous frits that seal the bottom of each well [21]. However, the reliability of this device is strictly dependent on the accuracy of plate manufacturing, in particular the porosity and sealing of the frits. Furthermore, in HT screening the device requires laborious handling to connect hundreds of well/tubing pairs. In these respects, the Air-Well minifermenter presented here offers several advantages: the 96 needles plate fits any commercially available 2 ml deep-well plate, with any section (round or squared) or bottom profile (U, V or flat); the assembly of the cultivation unit (i.e. one 96 needles plate clamped to a Plexiglas bell) is operationally simple and fast; the absence of filters and the negligible hydrostatic pressure of well fluid allow the use of a low pressure air pump. Nevertheless, when we built the first device, we faced two major problems: the air supply was not equal in each well, particularly at very low pressure, and the bubbles formed by continuous air flow did not dissipate efficiently, increasing the risk of well-to-well contamination. We solved these problems by three simple means. First we inserted three grids within the Plexiglas bell to increase the turbulence of air flow and equalize its diffusion (Figure 1C). Second, we decided to provide air as a pulsed flux rather than continuously. This was achieved by controlling the open/closed status of an in-line solenoid valve with a square wave generator. As a result we obtained an even distribution of air flow across the entire plate (Figure 1A and 3D). Finally, since we observed that the volume of bubbles and the time to dissipate increased with the increase of cell density, we decided to complement the medium with an antifoam reagent at a concentration that did not affect cell growth (Figure 2A,B). We provide comparative evidence demonstrating that *E. coli* cells grow faster and reach higher cell density when cultivated in the Air-Well minifermenter (Figure 3). Furthermore, the use of an autoinduction medium [9] allows cultivation in batch-mode without interruptions during culture growth. We are aware that, when compared to other miniaturized bioreactors (e.g. the Robo-Lector, [11]), our Air-Well minifermenter lacks the possibility of on-line monitoring of cultivation parameters, thus being unsuitable, in its existing format, to study cultivation processes in detail. However, we demonstrate that cultivation by the Air-Well minifermenter is *per se* sufficient to improve the quantity and quality of recombinant proteins produced by different clones (Figure 4). This is likely due not only to the increase in the biomass of the culture but mainly to the effects that air sparging exerts on metabolic processes, decreasing the likelihood of anaerobic metabolism and avoiding the accumulation of noxious metabolites known to impair bacterial survival and recombinant protein production [12]. The Air-Well minifermenter has a simple design and is easy to reproduce. Air flow can be provided by any source, even an aquarium pump, and can be eventually regulated by a manometer. The pulsed air flow is generated by a common solenoid valve controlled by a wave generator program. As a stationary device, it requires limited lab space and a battery of four cultivation units can be held in a bench-top thermostatic cabinet. Finally, protein processing can be significantly speeded up by the easy availability of microtiter-based liquid-handling tools or robotics. For these features, and for the shorter time needed to get a productive biomass, we believe that the device described here satisfies most of the requirements for HT screening of recombinant proteins. The system has been used in previous screening [15,22,23], and proven to be robust and reproducible.

## Conclusions

The High-Throughput Protein Production in parallel for structural and functional studies has fostered high throughput systems development. In this article we demonstrate the advantage of using a minifermenter system tailored to the 96 deep-well plate format. The device uses air sparging to support the parallelized growth of hundreds of individual cultures. *E. coli* clones grow faster and reach higher cell densities than by shaken culture. Furthermore, the quantity and quality of the recombinant proteins produced are significantly improved. The device is composed of separate components and the air sparging plate can be easily tailored on other microtiter plate formats such as the 24 deep-well plates. The system is powered by low pressure pulsed air flow and a three way valve permits the influx of oxygen to improve culture growth. The system has proven to be robust and reproducible for HT screening.

## Methods

### Design and fabrication of the Air-Well minifermenter

The air well minifermenter (Figure 1) consists of a computer station and an air dispersing unit. The first unit consists of a computer device (Agilent 33220A Function/ Arbitrary Waveform Generator, 20 MHz) connected to an air compressor as air source and an electric valve. The air arriving from the compressor passes through a manometer and passes to the electric valve that controls the parameters (period (10-5-2.5 s), amplitude (5 Vpp) and width (200 ms) of the pulse) of the air flux. The incubation chamber that accommodates the air dispersing unit, is connected to the electric valve by a silicon tube and is located adjacent to the controller device. The air passes through a further tube fixed on a homemade Plexiglas bell which is joined by two steel clamps to a stainless steel 96 needle plate inserted into the 96 well culture plate (96 deep-well, 2.2 mL, VWR). The 96 needle plate is made of a stainless steel plate 91 mm in width, 133 mm in length and 10 mm thick. The 96 stainless steel minitubes inserted in the plate are 57.5 mm in length with an exterior and an interior diameter of 2 and 0.5 mm respectively; the needles are fixed with an epoxy resin-based glue that allows plate sterilization at 120°C. Needle diameters and air flux intensity were optimized previously to allow a suitable air flux to aerate and mix the cultures without well leakages (data not shown).

Air diffusion and flux homogeneity to the 96 insuffling needles was obtained by the use of plastic grids inserted into the Plexiglas bell (Figure 1B/C); thanks to these grids air flux is split and directed equally to the 96 needles

### Microorganism and media

*Escherichia coli* DH5αF’ strain was used. The pGEX 4 T-1® (GE Healthcare) plasmid vector for the expression of recombinant protein was chosen and modified to introduce BssHII and NheI restriction sites in the MCS to allow subcloning of ORF (Open Reading Frame) fragments of 96 different genes. The ORF fragments were produced fused with a GST tag at the N-termini, and a sequence codifying for the 7 aa FLAG tag was introduced at the 3’ end to assess the production of a full-length recombinant protein and to investigate the degradation level. The growth and protein production experiments were carried out in the autoinduction media developed by F.W. Studier [9]. The medium had the following composition as final concentration: ZY (1% Tryptone, 0,5% Yeast extract); 25 mM Na2HPO4, 25 mM KH2PO4, 50 mM NH4Cl, 5 mM Na2SO4; 2 mM MgSO_4_; Metal Mix (50uM FeCl3, 20uM CaCl2, 10uM MnCl2, 10uM ZnSO4, 2uM CoCl2, 2uM CuCl2, 2uM CuCl2,2uM NiCl2, 2uM Na2MoO4, 2uM Na2SeO3, 2uMH3BO3); 5052 solution (0,5% glycerol, 0,05% glucose, 0,2% α-lactose monohydrate); 100 μg/mL of ampicillin, sterile water to volume. The medium was supplemented with 0.01% antifoam 204 (SIGMA) to avoid bubble formation in bacterial cultures.

### Growth conditions

If not otherwise specified, the experiments were conducted with 900 uL working volume of auto-inducing media, at 37°C constant temperature during the day and 28°C for overnight growth. Shaking cultures were grown at 200 rpm on an orbital shaker (IS-971R, Jeio Tech, England). For each experiment, all plates received the same inoculum prepared from a single colony. During the growth, an aliquot of each cell culture was harvested and a reference OD_600_ reading was taken with a spectrophotometer (VICTOR X Multilabel Plate Readers, PerkinElmer).

### Protein purification

For the protein extraction 100 μL of FastBreak® (Promega) lysis buffer was added to each well; the plate was then sealed with adhesive aluminium film and incubated for an additional 15 min with gentle shaking at 4°C. 10 μL of DNAse (10 mg/mL) were added in each well and the plate was incubated for 15 min with gentle shaking at 4°C, in order to reduce the viscosity of solution. Lysozyme (stock 20 mg/mL) was diluted 1:10 in water just before use and 10 μL were added in each well. The plate was then incubated for 20 min with gentle shaking at 4°C to complete clarification of lysates.

GSH magnetic beads (Promega) were prepared according to manufacturer instruction; the bacterial lysates were transferred to a 1 mL 96 deep-well plate (Costar) and 25 μL of the magnetic beads suspension were added to each well. The plate was then sealed with an aluminium film and incubated with gentle mixing on a rotating platform at 4°C for 30 min; particles were not allowed to settle for more than a few minutes, as this reduces binding efficiency. The plate was finally set on the magnetic support (V&P Scientific), so that the GSH particles were captured by the magnets. A 96 Tube Aspiration Manifold (V&P Scientific) connected to the vacuum pump was then used to carefully remove supernatant. The manifold was then removed, the plate taken off the magnetic stand, and 250 μL of binding/wash buffer (50 mM Tris–HCl pH7.5, 150 mM NaCl, 1% (v/v) Triton X-100) were added to each well with a multichannel pipette. The plate was gently mixed for 5 min at 4°C. The plate was placed back on the magnetic stand and washing procedure was repeated for 3 times.

Elution of the bound protein was performed by adding 25 μL of elution buffer (50 mM GSH, 100 mM NaCl, PBS, pH adjusted to 8.0) in each well; the plate was incubated with gentle mixing at 4°C for 15 min.

The plate was placed back on the magnetic stand, the supernatant was carefully removed with a multichannel pipette, and the eluted GST-fusion proteins were transferred into a new 96-well plate. A second elution step may be performed following the same procedure. The aliquots of the eluted protein were kept either at 4°C or, for longer storage, at −70°C (in this case, 10% glycerol was added).

### Microarray analysis

A BioOdissey Calligrapher (Biorad) equipped with 150 μm solid pins was used to print recombinant proteins onto nitrocellulose FAST® slides (Whatman). Humidity was set at 50%, temperature at 20°C. Proteins supplemented with 0,005% Triton X-100 were loaded 7 μL per well in 384 multi-well plates (Genetix) and printed as two replicate spots. Distance between adjacent spots was 560 μm.

After spotting, nitrocellulose slides were blocked with 3% non-fat dry milk in PBS-Tween20 0.1% for 1 h at RT. Incubation with primary antibodies 1:3000 (antiGST and antiFLAG, Sigma) were performed in 2% non-fat dry milk in PBS Tween20 0.1% at RT for 2 h with gentle agitation.

Slides were washed twice with PBS-Tween20 0.1% and then with PBS for 15’. Secondary antibodies (anti mouse Cy-5 conjugated, Jackson ImmunoResearch) were diluted 1:200 in 2% non-fat dry milk in PBS-Tween20 0.1% for 1 h at RT, then washed as mentioned before, rinsed with water, spun for 2’ at 1500 rpm and dried in the dark at RT.

Fluorescence was detected with a ScanArray Gx®, PerkinElmer scanner and analyzed with ScanArray Expression Software (PerkinElmer). The fluorescence intensity of each feature was determined, based on the mean of pixels’ intensity within the protein feature minus the mean intensity of pixels in the surrounding area.
